# Menstruation in Old Age

**Published:** 1856-12

**Authors:** 


MENSTRUATION IN OLD AGE.—J. J. Dixon, M. D., of Ashland, Tennessee, in a letter to the Editors of the *Atlanta* (Ga.) *Medical Journal*, gives the following interesting item:

" In a few lines, I wish to record a brief account of a singular case to which I was this day called. The patient was an old lady, age 67, who is now menstruating. She is the mother of eight children; her menses ceased nineteen years ago, since which time she has enjoyed respectable health. Menstruation returned eleven months since, and has now occurred, in all, six times. She has not suffered any serious difficulty until the present period, and her symptoms now seem only to be those attendant upon painful menstruation."

